# Digital Health Communication and Vaccine Confidence in Mexico Using Aggregated Randomized Brand Lift Studies: Secondary Analysis

**DOI:** 10.2196/82889

**Published:** 2026-01-06

**Authors:** Berenice Muñoz Cordero, Rodrigo Romero Feregrino, Raul Romero Feregrino, Raúl Romero Cabello, Valeria Magali Rocha Rocha, Roberto Martinez-Medina, Liliana Aline Fernández Urrutia

**Affiliations:** 1 Asociación Mexicana de Vacunología, Mexico City, Mexico; 2 Instituto Para el Desarrollo Integral de la Salud (IDISA), Av. Cuauhtemoc 271 int. 101, Col. Roma, Alc. Cuauhtémoc, Mexico City, 06700, Mexico, 52 5555840843; 3 Department of Pediatrics, Hospital General de Cuajimalpa IMSS-Bienestar, Mexico City, Mexico; 4 Saint Luke School of Medicine, Mexico City, Mexico; 5 Employer Sector CONCAMIN, Technical Council, Instituto Mexicano del Seguro Social, Mexico City, Mexico; 6 Academia Mexicana de Pediatria, Mexico City, Mexico; 7 Department of Pediatric Infectology, Pediatrics Hospital “Dr. Sylvester Frenk Freund” 21st Century National Medical Center, Instituto Mexicano del Seguro Social, Mexico City, Mexico; 8 Department of Infectology, Hospital General de Mexico, Mexico City, Mexico; 9 Department of Microbiology and Parasitology, Universidad Nacional Autónoma de México, Mexico City, Mexico; 10 Life Sciences and Health Sciences, Universidad Popular Autónoma del Estado de Puebla, Puebla, Mexico

**Keywords:** COVID-19 vaccine, health behavior, health communication, social media, vaccination coverage, Mexico, health promotion, vaccine confidence, Bayesian analysis, digital health interventions, vaccine acceptance, public health messaging

## Abstract

**Background:**

Digital vaccination campaigns are increasingly used to address declining vaccine confidence, yet evidence from large-scale, real-world interventions in middle-income countries is limited. Meta’s Brand Lift Studies (BLS), which use randomized test-control exposure, provide Bayesian estimates of attitudinal shifts resulting from digital content. Mexico, with over 88.6 million active internet users, provides a setting to evaluate the impact of targeted campaigns on vaccine attitudes.

**Objective:**

This study evaluated the impact of 5 digital vaccination campaigns implemented by the Asociación Mexicana de Vacunología (@Vacunologia) on Facebook (Meta Platforms Inc) and Instagram (Meta Platforms Inc) in Mexico between 2021 and 2022 on key attitudinal constructs related to COVID-19 vaccine confidence.

**Methods:**

This study used a retrospective ecological design. We analyzed aggregated BLS results for 5 campaigns targeting different audiences and vaccination themes. Measured outcomes included standard ad recall, perceived importance, perceived safety, perceived efficacy, and concerns about side effects. Statistical significance within the BLS framework was defined as an incremental lift of ≥2 percentage points with ≥90% posterior probability of replication—a threshold consistent with Meta’s operational Bayesian approach. Exploratory comparisons across campaigns were conducted using 1-way ANOVA, unpaired 2-tailed *t* tests, and Fisher exact tests.

**Results:**

Campaigns reached 84.9 million accounts and generated 179.4 million impressions with a total investment of US $215,600. All campaigns produced statistically significant improvements in at least one attitudinal outcome (Bayesian threshold ≥90%). Standard ad recall increased in 4 campaigns (ANOVA, *P*<.001), and concerns about side effects decreased in 2 campaigns (*t* test, *P*=.049; *P*=.006). Perceived safety, importance, and efficacy improved in selected audiences, with stronger effects observed among younger users and women (ANOVA, *P*=.005). No direct behavioral outcomes (eg, vaccination uptake) were measured; therefore, the findings reflect attitudinal rather than behavioral change. However, these constructs are recognized as proximal predictors of vaccine decision-making and constitute health-related outcomes.

**Conclusions:**

Large-scale digital vaccination campaigns can meaningfully strengthen attitudinal determinants of vaccine confidence in a middle-income context. These social media advertising campaigns effectively increased standard ad recall and improved perceptions of vaccine importance and safety, particularly among younger audiences and women in urban areas. However, changes in efficacy perceptions and concerns about side effects were limited. The innovation and implications of this study lie in evaluating large-scale, real-world digital vaccine campaigns in Latin America using experimental BLS data. Findings highlight that audience segmentation yields stronger perceptual shifts, suggesting that tailored digital strategies can complement traditional public health communication. While the BLS does not measure behavioral end points, the observed attitudinal improvements represent foundational steps toward influencing vaccine-related behaviors. Future work should link digital attitudinal metrics with vaccination and epidemiological data to assess real-world health impact.

## Introduction

Vaccination is one of the greatest achievements of the 20th century, yet vaccine hesitancy and delayed uptake remain persistent public health challenges [1]. In March 2020, the World Health Organization (WHO) declared COVID-19 a pandemic, and the approval of the first vaccines in December 2020 was expected to mitigate its impact [2]. However, ensuring widespread acceptance required effective communication strategies to address doubts and misinformation.

During the pandemic, social media emerged as a critical channel for rapidly disseminating health information, enabling governments and organizations to reach large audiences with targeted messages [3]. Globally, more than 3.5 billion people use social media, spending an average of 3 hours per day on these platforms [4]. In Mexico, in 2021, there were 88.6 million internet users (75.6% penetration) of the population aged 6 years or older [5]. This makes Mexico a unique setting to evaluate the effectiveness of digital campaigns in shaping vaccine-related perceptions.

Evidence on digital health interventions suggests that campaigns with greater frequency, interactivity, and feedback are more effective in influencing attitudes and behaviors [4]. A Cochrane review of interventions to increase COVID-19 vaccine acceptance found heterogeneous strategies, with communication interventions being the most common, but highlighted gaps in studies from low- and middle-income countries (LMICs) [2]. Similarly, a meta-analysis of over 800 digital public health experiments showed that online advertising can shift beliefs and attitudes about COVID-19 and may represent a cost-effective approach to increasing vaccination rates [3].

Digital health communication on social media exerts a complex and multifaceted impact on vaccine confidence, with both positive and negative effects documented in the medical literature.

Large-scale randomized experiments of public health social media campaigns targeting COVID-19 vaccination have demonstrated that such interventions can modestly improve vaccine-related beliefs and knowledge, with measurable effects on self-reported attitudes and behaviors. For example, targeted social media advertising was shown to shift vaccine-related opinions and increase knowledge about vaccine access, with an estimated cost-effectiveness for influencing vaccination rates [3]. Systematic reviews of randomized controlled trials further support the potential for social media platforms to disseminate quality-assured information and increase vaccine uptake, but emphasize that the overall effect is variable and often nonsignificant, highlighting the need for careful moderation and integration with other communication strategies [6].

Conversely, observational and qualitative studies consistently report that social media is a major vector for the spread of vaccine misinformation and hesitancy. Thematic analyses of social media content and epidemiological survey responses reveal that social media interactions can perpetuate distrust in vaccine safety, skepticism about efficacy, and ideological isolation, particularly when algorithms amplify echo chambers and misinformation [7-9]. Heavy reliance on social media as a primary news source is associated with increased vaccine hesitancy, especially regarding beliefs that COVID-19 risks are exaggerated or that vaccines are unsafe [8,9]. The influence of social media is especially pronounced among individuals with lower news literacy, who are more susceptible to skepticism and hesitancy [9].

The impact of social media varies by platform and user characteristics. For instance, among mothers of adolescents, uncertainty about the veracity of social media messages is associated with increased human papillomavirus vaccine hesitancy, while Facebook (Meta Platforms Inc)-specific influence was negatively associated with hesitancy, suggesting that platform-specific dynamics and content moderation may play a role [10]. Additionally, unvaccinated individuals are more likely to report social media and messenger services as persuasive sources compared to vaccinated individuals, underscoring the importance of targeted science communication to counteract misinformation [11].

Social media analytics have also been shown to correlate with real-world vaccine uptake rates, suggesting that monitoring online sentiment can provide actionable insights for public health interventions, even if self-reported vaccine acceptance does not always align with actual behavior [12].

Despite the rapid expansion of digital communication during the COVID-19 pandemic, very few studies have evaluated the real-world impact of large-scale social media campaigns on vaccine perceptions in LMICs. Most existing evidence comes from high-income settings, leaving a gap in understanding how digital interventions perform in diverse populations with varying levels of vaccine hesitancy and information access. Mexico, with its high social media penetration and persistent challenges in vaccine confidence, represents a critical context to examine whether targeted digital campaigns can shift perceptions related to vaccination. This study addresses that gap by analyzing experimental Brand Lift data, offering empirical evidence from a region where such evaluations remain scarce.

This study addresses these gaps by evaluating 5 large-scale social media advertising campaigns conducted by the Asociación Mexicana de Vacunología (@Vacunologia) on Facebook and Instagram (Meta Platforms Inc) between 2021 and 2022. Using anonymized, aggregate-level data from Meta’s Brand Lift Studies (BLS), we assess the campaigns’ impact on standard ad recall and on perceptions of the importance, safety, efficacy, and side effects of COVID-19 vaccines. Importantly, this study does not measure actual vaccination uptake, but rather attitudinal and perceptual shifts that serve as precursors to behavior. By analyzing real-world campaigns in a middle-income country, this research contributes novel evidence on the role of digital media in public health communication in Latin America.

Decision-making regarding vaccines and other health topics is complex and influenced by multiple factors such as personal beliefs, experiences, and external influences. Effectively shifting attitudes toward vaccines requires sustained communication efforts across various levels—personal, community, and institutional.

Therefore, the objective of this study is to evaluate the effectiveness of 5 social media advertising campaigns on COVID-19 vaccination conducted in Mexico through Facebook and Instagram. Specifically, we aim to assess their impact on standard ad recall, perceived importance, safety, efficacy, and concerns about side effects, and to determine which audiences (by age and sex) were most responsive. Our working hypothesis is that targeted campaigns, particularly those directed at younger populations and women, would generate stronger positive shifts in vaccine-related perceptions compared to general population campaigns.

## Methods

### Research Design

We conducted a retrospective ecological analysis using anonymized, aggregated data from 5 BLS implemented by Meta on Facebook and Instagram. BLS are randomized ad-exposure experiments embedded within the social media platform, where users are automatically assigned to test (exposed) or control (unexposed) conditions. Because no individual-level longitudinal data, identifiers, or person-level outcomes are produced, this study does not constitute a retrospective cohort or case-control design. All analyses were performed using the aggregate outputs supplied by Meta.

### Participants

All BLS results are aggregated and anonymized. BLS results for Meta are disaggregated by age and sex. Each audience member is likely to be presented with only 1 question in their newsfeed. This implies that all creative content should align with the BLS questions, as there is no control over which creative or question they will encounter. Facebook considers any Brand Lift result that shows a 2 percentage point or higher increase with at least a 90% probability of a Brand Lift to be statistically significant. Below are several questions to consider if a Brand Lift result greater than 2 percentage points is observed.

For the implementation of the BLS programs, an agreement was signed for the “Scaled Program Support” Program Participation between Facebook Inc and Asociación Mexicana de Vacunología.

The results provided by Facebook do not contain personal information about the users; they are only statistics about the BLS as presented in Tables 1-3, which were used to obtain the statistical results. The data were obtained by the BLS tools of Facebook Inc, in accordance with its privacy policies [13]. Table 1 summarizes problem statements, behavior change and communication goals, target audiences, survey questions, advertising objectives, campaign duration, and investment.

**Table 1.** Characteristics of 5 Brand Lift Studies to promote COVID-19 vaccination in Mexico, 2021‐2022.

Category	Campaign 1	Campaign 2	Campaign 3	Campaign 4	Campaign 5
Problem statement	There are a large number of people in Mexico who are still undecided about getting a COVID-19 vaccine.	According to recent data from the Data for Good survey, there are a large number of women in Mexico who are still undecided about getting a COVID-19 vaccine.	There are people who travel in Mexico who are still undecided to take the COVID-19 vaccine booster.	There are people in Mexico who have doubts about the effectiveness and safety of the COVID-19 vaccine booster.	There are people in Mexico who have doubts about the COVID-19 vaccine booster if it works, especially to prevent serious diseases.
Behavior change goal	Get the COVID-19 vaccine	Get the COVID-19 vaccine	Get the COVID-19 vaccine booster	Get the COVID-19 vaccine booster	Get the COVID-19 vaccine booster
Communications goal	Decrease COVID-19 vaccine hesitancy in Mexico	Increase confidence in vaccination	Increase confidence in vaccination	Increase confidence in vaccination	Increase confidence in vaccination
Campaign target audience	Age range: >18 Gender: All Geography: All of Mexico	Age range: 18‐44, Gender: Women Geography: Ciudad de México; Puebla; Guadalajara, Monterrey	Age range: >18 Gender: All Geography: All of Mexico	Age range: >18 Gender: All Geography: All of Mexico	Age range: >18 Gender: All Geography: All of Mexico
Questions	Recall: Do you recall seeing an ad for COVID-19 vaccines from @Vacunologia online or on a mobile device in the past 2 days? Importance: How important do you feel a vaccine is to prevent COVID-19? Safety: How safe do you think a COVID-19 vaccine is for people like you?	Recall: Do you recall seeing an ad for COVID-19 vaccines from @Vacunologia online or on a mobile device in the past 2 days? Side effects: How concerned are you about the potential side effects of a COVID-19 vaccine? Safety: How safe do you think a COVID-19 vaccine is for people like you?	Recall: Do you recall seeing an ad for COVID-19 vaccines from @Vacunologia online or on a mobile device in the past 2 days? Safety: How safe do you think a COVID-19 vaccine is for people like you? Importance booster: How important do you think the booster dose of the vaccine is to protect against COVID-19? Efficacy: How effective do you think a COVID-19 vaccine booster is in protecting against COVID-19?	—^a^	—^a^
Campaign advertising objective	Reach	Page likes	Reach	Reach	Reach
Duration	June 6 to June 30, 2021	September 10 to October 5, 2021	June 16 to August 7, 2022	June 16 to August 7, 2022	June 16 to August 7, 2022

a These questions are also applicable to campaigns 4 and 5.

**Table 2.** Results of 5 Brand Lift Studies evaluating COVID-19 vaccination campaigns on Facebook and Instagram in Mexico, 2021‐2022.

BLS^a^ metrics	1	2	3	4	5
Campaign advertising objective	Reach	Page likes	Reach	Reach	Reach
Duration (days)	22	25	54	54	54
Investment (US $)	14,800	14,920	58,708	58,708	68,493
Reach	18.3 million	1.9 million	21.5 million	21.4 million	21.6 million
Average frequency	2.4	7.7	1.7	1.7	1.7
Impressions	44.7 million	15 million	37.2 million	37.1 million	45.1 million
CPM^b^ (US $)	0.3	0.9	1.5	1.5	1.4
Standard ad recall	2.1	11.6	3.8	2.6	−0.6
Importance	2.6	—^c^	2.7	1.4	3.2
Safety	0.3	2.4	−1.1	1.3	1.5
Side effects	—	−0.6	—	—	—
Efficacy	—	—	0.9	1.1	1

a BLS: Brand Lift Studies.

b Cost per thousand impressions.

c Not applicable.

**Table 3.** Results of 5 Brand Lift Studies on COVID-19 vaccination campaigns in Mexico, 2021‐2022, stratified by sex and age group.

Sex and age group	Female, 18‐24	Male, 18‐24	Female, 25‐34	Male, 25‐34	Female, 35‐44	Male, 35‐44	Female, 45‐54	Male, 45‐54	Female, 55‐64	Male, 55‐64	Female, ≥65	Male, ≥65
Campaign 1												
Standard ad recall	+1.3	+5.9^a^	+2.6	−2.0	−0.6	+5.9^b^	+2.5	+3.0	+3.0	−2.1	+3.8	+6.0^b^
Importance	+2.6^b^	+1.5	+5.3^a^	+4.0^a^	+3.0^b^	+2.5^b^	+0.0	+1.2	+0.6	−0.8	−0.5	+0.5
Safety	−1.0	−1.6	−0.7	+3.2^b^	+1.1	+0.2	−1.0	+1.1	−0.0	+0.3	+1.0	+0.2
Campaign 2												
Standard ad recall	+6.7^a^	—^c^	+13.1^a^	—	+12.7^a^	—	—	—	—	—	—	—
Side effects	−0.4	—	+0.3	—	−2.4	—	—	—	—	—	—	—
Safety	+3.4^a^	—	+2.5^b^	—	+1.5	—	—	—	—	—	—	—
Campaign 3												
Standard ad recall	+3.2^b^	+5.0^a^	+3.7^b^	+2.6	+5.1^b^	+3.9^b^	—	—	—	—	—	—
Safety	−1.3	−0.7	+1.4	−0.7	−4.0	−3.3	−2.6	+0.1	—	—	—	—
Importance	+5.0^a^	+0.8	+5.5^a^	−0.6	+0.9	+4.6^a^	+1.0	+1.4	—	—	—	—
Efficacy	+2.5^b^	+3.1^b^	+0.1	+1.9	−4.4	+0.1	+1.4	+0.8	—	—	—	—
Campaign 4												
Standard ad recall	+5.0^a^	−3.5	+5.6^a^	+2.4	+2.1	+3.5	+4.9	—	—	—	—	—
Safety	+1.2	−2.4	+5.1^a^	+3.2^b^	+0.1	−1.6	−0.4	+1.2	—	—	—	—
Importance	+3.3^a^	+3.4^b^	−0.7	+1.4	+1.7	−1.2	−0.3	+1.1	—	—	—	—
Efficacy	+0.1	+0.5	+3.8^a^	+3.1^b^	−3.8	+1.2	+1.3	−3.6	—	—	—	—
Campaign 5												
Standard ad recall	−1.2	+1.6	−1.9	−0.4	−0.2	−2.2	+2.2	+3.6	—	—	—	—
Safety	+4.9^a^	−2.7	+3.5^a^	−0.3	+1.1	+2.5	+0.8	+1.5	—	—	—	—
Importance	+4.9^a^	+1.0	+3.8^a^	+3.9^a^	+4.0^a^	+0.4	+3.4	+1.2	—	—	—	—
Efficacy	+1.3	+2.3	+0.5	+3.7^a^	−1.6	−2.3	−0.7	+1.7	—	—	—	—

a 90% or greater chance that the increase is caused by Brand Lift.

b 80% or greater chance that the increase is caused by Brand Lift.

c Not applicable.

The Asociación Mexicana de Vacunología A.C. accepted and approved the use of the results of the BLS performed for the realization of this study.

The Asociación Mexicana de Vacunología (@Vacunologia) participated in Facebook’s 2021 and 2022 Scaled Workshop Social and Behavior Change Communication (SBCC) Program. As part of this program, the Asociación Mexicana de Vacunología received Facebook Ad Credits and support to conduct a public health campaign on Meta. The campaign characteristics are detailed in Table 1.

We analyzed information from 5 BLS conducted by the Asociación Mexicana de Vacunología on Facebook and Instagram (@Vacunologia). BLS are experimental tools developed by Meta to evaluate the impact of advertising campaigns independently of other marketing activities. They work by randomly dividing a representative sample of eligible users into 2 groups: a test group, which is exposed to the ads, and a control group, which is not. Both groups are then asked standardized poll questions, and the difference in responses indicates the incremental effect, or “lift,” attributable to the campaign [14].

Randomization and audience segmentation are performed automatically by Meta’s platform, using predefined targeting criteria such as age, sex, location, or interests. This design ensures internal validity and allows causal inference about the effect of ad exposure [15]. Lift is reported as the percentage point difference between the test and control groups, accompanied by a confidence estimate. Outcomes typically include awareness, recall, or perceptions related to the advertised content.

The study population consisted of adult Facebook and Instagram users in Mexico who were eligible to be exposed to the campaigns according to predefined targeting criteria (eg, all adults nationwide, or women aged 18‐44 years in major cities). Randomization was performed automatically by Meta’s BLS platform at the user ID level, assigning individuals to either the test group (with the opportunity to see the ads) or the control group (withheld from exposure). This design ensured balance between groups and minimized systematic differences at baseline. The only demographic variables available for analysis were age group and sex, which were provided in disaggregated form in the BLS reports. Because the data were anonymized and aggregated, no additional covariates were accessible.

### Sampling Procedures

#### Overview

There are different types of lift tests: Conversion Lift (sales, leads, and engagement), Brand Lift (awareness and attitudes), and Experiments (custom questions on brand or campaign objectives) [16]. In our case, the BLS measured outcomes relevant to vaccination, such as standard ad recall, perceived importance, safety, efficacy, and concerns about side effects. These results—whether positive or negative—provide valuable insights into the effectiveness of digital communication strategies for vaccines.

In this study, the conversion rate was defined as the proportion of respondents in each group who provided a positive response to a BLS survey item. The expected conversion rate was estimated from the control group’s responses, representing the baseline that would have been observed in the test group without ad exposure. Lift was calculated as the difference between the observed conversion rate in the test group and the expected rate from the control group, with ≥2 percentage points and ≥90% probability considered statistically significant.

Lift is widely used because it offers a standardized way to compare campaigns across contexts. Formally, it represents the relative change in outcomes among exposed users compared to the expected rate had they not been exposed. However, when baseline rates are very low, even small absolute differences can produce large lift values, which may overstate effectiveness. Despite this limitation, lift remains an informative indicator of advertising performance, particularly in digital public health interventions where direct behavioral outcomes (eg, vaccination uptake) are difficult to measure [17].

The BLS survey included 2 types of questions depending on the construct being evaluated. The recall question offered 3 response options: Yes, No, and I am not sure. In contrast, questions assessing perceived importance, safety, side effects, and efficacy were based on 5-point Likert-type scales. For example, the safety perception item included the options: Very safe, Somewhat safe, Slightly safe, Not safe, and I don’t know. Depending on the specific construct, the term “safe” was replaced with “important,” “effective,” or the corresponding attribute. Changes in responses were categorized as either positive or negative shifts in perception, depending on whether the response moved toward or away from more favorable attitudes (eg, from “Somewhat safe” to “Very safe” vs from “Very safe” to “Not safe”). These directional changes were interpreted as proximal indicators of positive or negative behavior change, consistent with established frameworks in behavioral science.

#### Measures

The Meta reports use the Basic Bayesian Lift methodology, which provides estimates of confidence rather than *P* values. The Basic Bayesian results closely resemble frequentist results but offer confidence estimates. The observed difference between the control and test groups, not attributed to chance but instead attributed to a greater than 90% chance of Brand Lift being observed, is considered to have high confidence. If the ad were shown to a random sample of the target audience, there would be a 90% likelihood of observing the same lift from the control to the test group.

Several factors are taken into consideration when determining the chance of Brand Lift being observed, including the number of respondents answering the question (test group sample size), control response rate (control group sample size, often referred to as “base”), and the size of the lift (difference between control and test group scores). As the sample size increases for both groups, the scores become more stable. With more stable scores, smaller differences can be confidently declared as statistically significant.

The Bayesian statistical method is generally equivalent to frequentist statistical methods in most cases but offers a more conservative measure of confidence when there are known sources of bias or systematic errors. Therefore, observing any statistically significant positive shift (a “lift” of 2 percentage points or more) in vaccine attitudes as a result of an online campaign alone is a significant achievement and worthy of celebration.

In this study, we present the results provided by the Meta company. The outcomes measured were: (1) standard ad recall, (2) perceived importance of vaccination, (3) perceived safety, (4) perceived efficacy, and (5) concerns about side effects. These were assessed through BLS poll questions. No direct behavioral outcomes (eg, actual vaccination uptake) were available. Because Meta’s BLS provide only aggregated, anonymized summary data, the dataset contained no item-level missing data. Each respondent is randomly assigned a single survey question, meaning that the number of observations varies across constructs and demographic subgroups by design. Therefore, the smaller n’s observed in certain cells reflect the structure of the BLS survey, not missing or incomplete responses.

### Analytic Strategy

Statistical analyses included 1-way ANOVA to compare mean scores across campaigns, unpaired *t* tests for sex differences, and Fisher exact tests to examine associations between campaign characteristics and the probability of achieving a Brand Lift (≥80% or ≥90% confidence). Bayesian confidence estimates from Meta’s BLS are not equivalent to frequentist *P* values. Frequentist tests (ANOVA, *t* tests, and Fisher exact tests) were used only for comparisons derived from the summary metrics provided in Tables 2-7.

**Table 4.** Comparison of mean scores (with SDs) across 5 Brand Lift Studies of COVID-19 vaccination campaigns in Mexico, 2021‐2022.

Outcomes	1, mean (SD)	2, mean (SD)	3, mean (SD)	4, mean (SD)	5, mean (SD)	*P* value^a^
Standard ad recall	2.4 (2.9)	10.8 (3.6)	3.9 (1.0)	2.9 (3.1)	0.2 (2.1)	<.001
Safety	0.2 (1.3)	2.5 (1.0)	−0.9 (1.5)	0.8 (2.5)	1.4 (2.3)	.049
Efficacy	—^b^	—^b^	0.7 (2.3)	0.3 (2.8)	0.6 (2.0)	.95
Importance	1.7 (1.9)	—^b^	2.3 (2.3)	1.1 (1.7)	2.8 (1.7)	.29
Side effects	—^b^	−0.8 (1.4)	—^b^	—^b^	—^b^	—^b^

a *P* value from 1-way ANOVA*.*

b Not applicable.

**Table 5.** Comparison of Brand Lift Studies results by age group for COVID-19 vaccination campaigns in Mexico, 2021‐2022.

Age group (years), mean (SD)	18‐24	25‐34	35‐44	45‐54	55‐64	≥65	*P* value^a^
Standard ad recall	2.7 (3.4)	2.9 (4.6)	3.4 (4.4)	3.2 (1.1)	0.5 (3.6)	4.9 (1.6)	.90
Safety	0.0 (2.6)	1.9 (2.1)	0.1 (1.7)	0.1 (1.4)	0.2 (0.2)	0.6 (0.6)	.34
Efficacy	1.6 (1.2)	2.2 (1.6)	−1.8 (2.2)	0.2 (2.0)	—^b^	—^b^	.005
Importance	2.8 (1.6)	2.8 (2.5)	2.0 (1.9)	1.1 (1.1)	−0.1 (1.0)	0.0 (0.7)	.10
Side effects	−0.4 (0.0)	0.3	−2.4	—^b^	—^b^	—^b^	—^b^

a *P *value from 1-way ANOVA.

b Not applicable.

**Table 6.** Comparison of Brand Lift Studies results by sex for COVID-19 vaccination campaigns in Mexico, 2021‐2022.

Variable	Male, mean (SD)	Female, mean (SD)	*P* value^a^
Standard ad recall	2.2 (3.1)	3.7 (4.0)	.06
Safety	−0.1 (1.9)	1.1 (2.0)	.13
Efficacy	1.3 (1.7)	−0.2 (2.6)	.21
Importance	1.5 (1.8)	2.4 (2.1)	.21
Side effects	—^b^	−0.8 (1.4)	.18

a Unpaired *t* test.

b Not applicable.

**Table 7.** Association between probability of behavior change (≥80% or ≥90% chance of Brand Lift) and campaign characteristics in 5 Brand Lift Studies of COVID-19 vaccination campaigns in Mexico, 2021‐2022.

Probability of Brand Lift	BLS, n (%)					*P* value^a^
	1	2	3	4	5	
Standard ad recall of 90% or more						.006
Yes	1 (8.3)	3 (100)	1 (12.5)	2 (25)	0 (0)	
No	11 (91.7)	0 (0)	7 (87.5)	6 (75)	8 (100)	
Standard ad recall of 80% or more						.03
Yes	2 (16.7)	0 (0)	4 (50)	0 (0)	0 (0)	
No	10 (83.3)	3 (100)	4 (50)	8 (100)	8 (100)	
Importance of 90% or more						.31
Yes	2 (16.7)	0 (0)	3 (37.5)	1 (12.5)	4 (50)	
No	10 (83.3)	0 (0)	5 (62.5)	7 (87.5)	4 (50)	
Importance of 80% or more						.22
Yes	3 (25)	0 (0)	0 (0)	1 (12.5)	0 (0)	
No	9 (75)	0 (0)	8 (100)	7 (87.5)	8 (100)	
Safety of 90% or more						.15
Yes	0 (0)	1 (33.3)	0 (0)	1 (12.5)	2 (25)	
No	12 (100)	2 (66.7)	8 (100)	7 (87.5)	6 (75)	
Safety of 80% or more						.41
Yes	1 (8.3)	1 (33.3)	0 (0)	1 (12.5)	0 (0)	
No	11 (91.7)	2 (66.7)	8 (100)	7 (87.5)	8 (100)	
Efficacy of 90% or more						>.99
Yes	0 (0)	0 (0)	0 (0)	1 (12.5)	1 (12.5)	
No	0 (0)	0 (0)	8 (100)	7 (87.5)	7 (87.5)	
Efficacy of 80% or more						.75
Yes	0 (0)	0 (0)	2 (25)	1 (12.5)	0 (0)	
No	0 (0)	0 (0)	6 (75)	7 (87.5)	8 (100)	
Side effects of 90% or more						
Yes	0 (0)	0 (0)	0 (0)	0 (0)	0 (0)	
No	0 (0)	3 (100)	0 (0)	0 (0)	0 (0)	
Side effects of 80% or more						
Yes	0 (0)	0 (0)	0 (0)	0 (0)	0 (0)	
No	0 (0)	3 (100)	0 (0)	0 (0)	0 (0)	

a Fisher exact test.

Bayesian confidence estimates from Meta’s BLS reflect the probability of observing the same incremental lift in a repeated randomized sample, and therefore should not be interpreted as frequentist measures of statistical significance.

### Ethical Considerations

This study was based exclusively on secondary, anonymized, and aggregate data provided by Meta’s BLS platform and did not involve any direct interaction or intervention with human participants. In accordance with national and international ethical guidelines, including the Declaration of Helsinki and the Council for International Organizations of Medical Sciences (CIOMS) International Ethical Guidelines, this type of research does not require formal review or approval by an institutional ethics committee. The Asociación Mexicana de Vacunología, as the coordinating institution, authorized the use of these data for academic and research purposes.

Because no individual-level data were accessed and no direct interaction with participants occurred, informed consent was not required. The original data collection by Meta was conducted under its own user agreements and privacy policies, which include provisions for the use of anonymized, aggregate data for research purposes. All data analyzed were anonymized and aggregated prior to researcher access. No personally identifiable information was available to the research team. Data were handled in accordance with Meta’s privacy policies and the Asociación Mexicana de Vacunología’s internal data protection standards.

No participants were recruited directly for this study, and therefore, no compensation was provided. No images or supplementary materials containing identifiable individuals are included in this paper.

### Reporting Guidelines

This study adheres to the APA Journal Article Reporting Standards for Quantitative Research (JARS-Quant), which provide structured guidelines for reporting research design, participant characteristics, measures, and analytic procedures. We followed the recommendations outlined in the APA JARS-Quant Table 1 to organize the “Methods” section and ensure transparent and comprehensive reporting of quantitative analyses [18].

## Results

Table 2 summarizes the results of the reports from the 5 BLS provided by the Meta company. Table 3 displays the results of the campaigns from the 5 BLS by sex and age group. It also indicates those that are likely to result in a change in behavior due to the campaign. Where we observe in black that the possibility of the increase being caused by the Brand Lift is 90% or greater and in gray 80% or greater.

**Figure 1.**
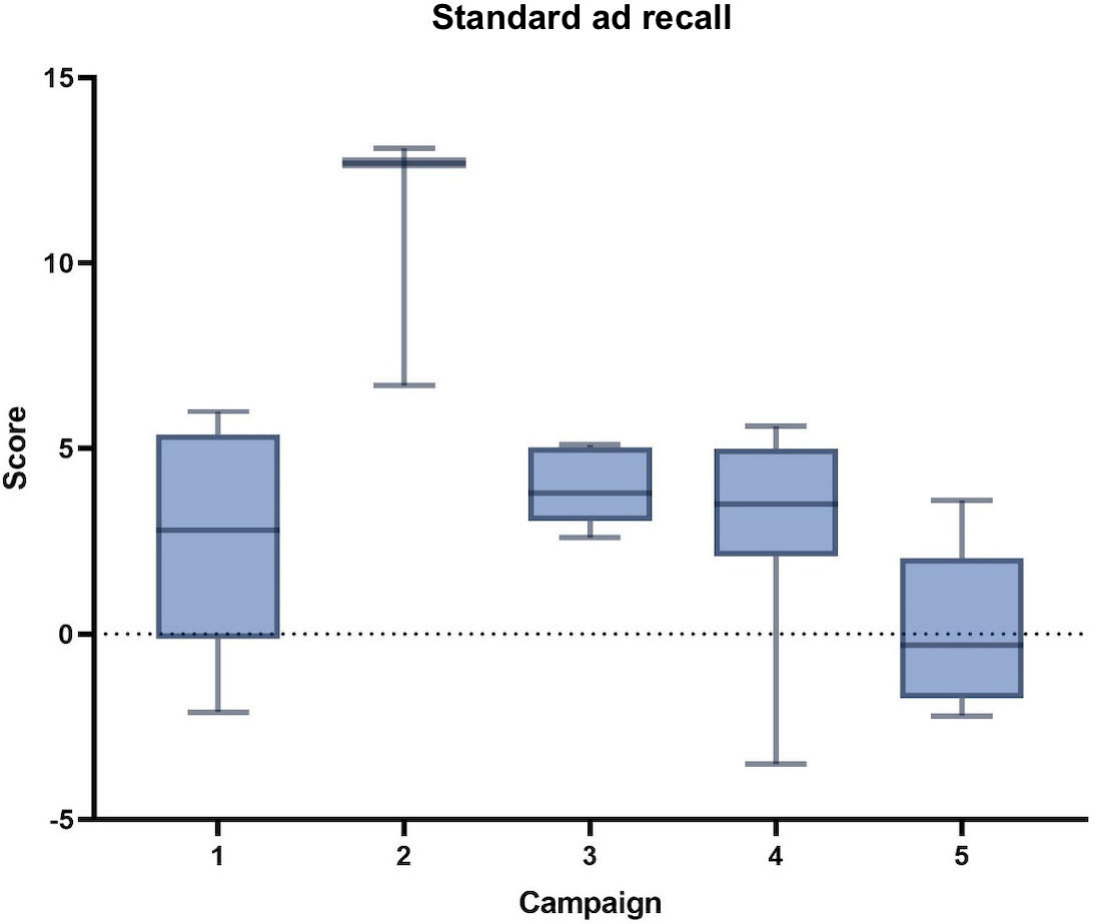
Incremental lift in standard ad recall across 5 Brand Lift Studies of COVID-19 vaccination campaigns conducted on Facebook and Instagram in Mexico, 2021‐2022.

The mean and SD are presented in the following table. A statistically significant difference was found in the mean of the standard ad recall (*P*<.001) and in the mean of the safety score (*P*=.049) between the campaigns as shown in Figures1  2. The comparison of scores by BLS was conducted, and the mean and SD are presented in Table 4.

**Figure 2.**
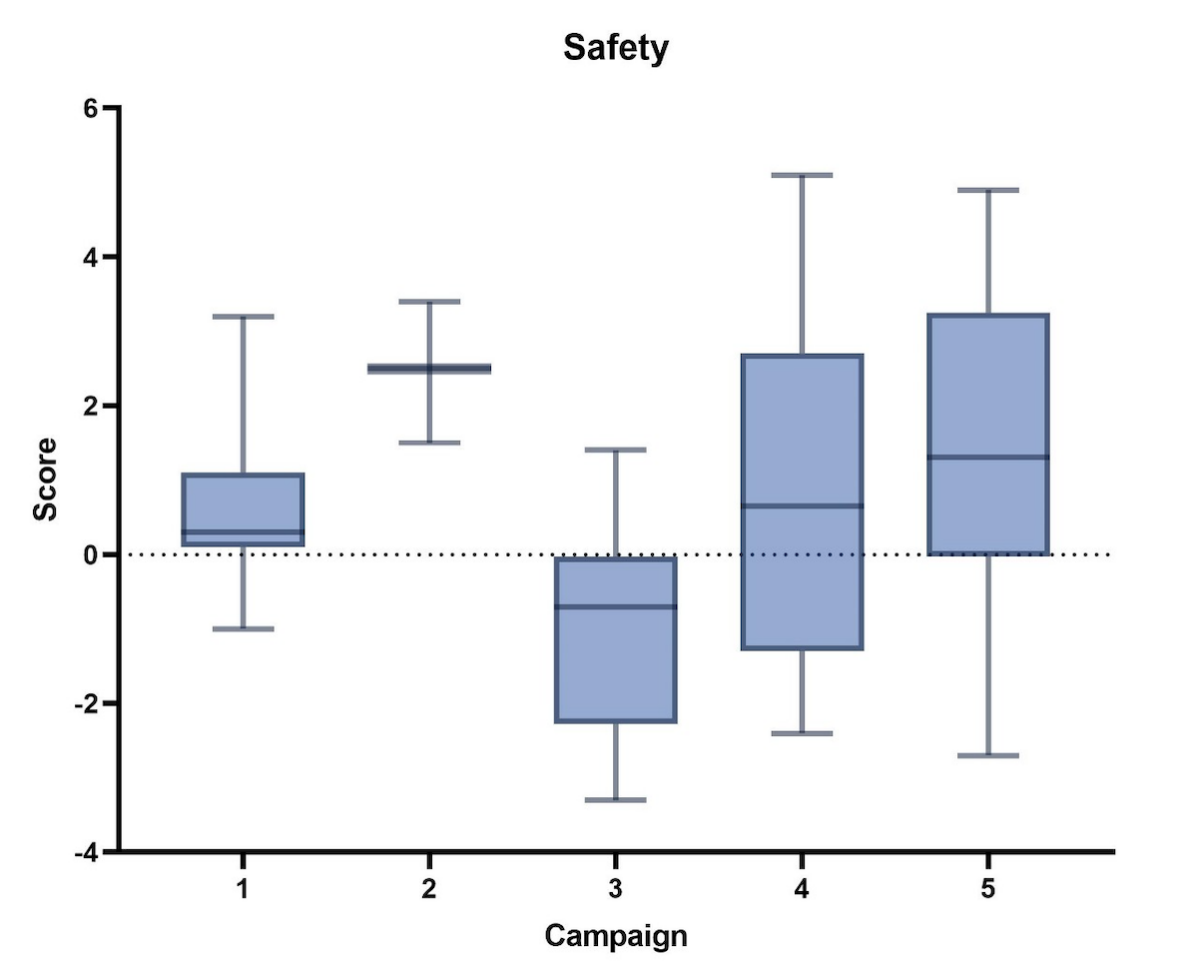
Incremental lift in perceived safety of COVID-19 vaccines across 5 Brand Lift Studies in Mexico, 2021‐2022.

**Figure 3.**
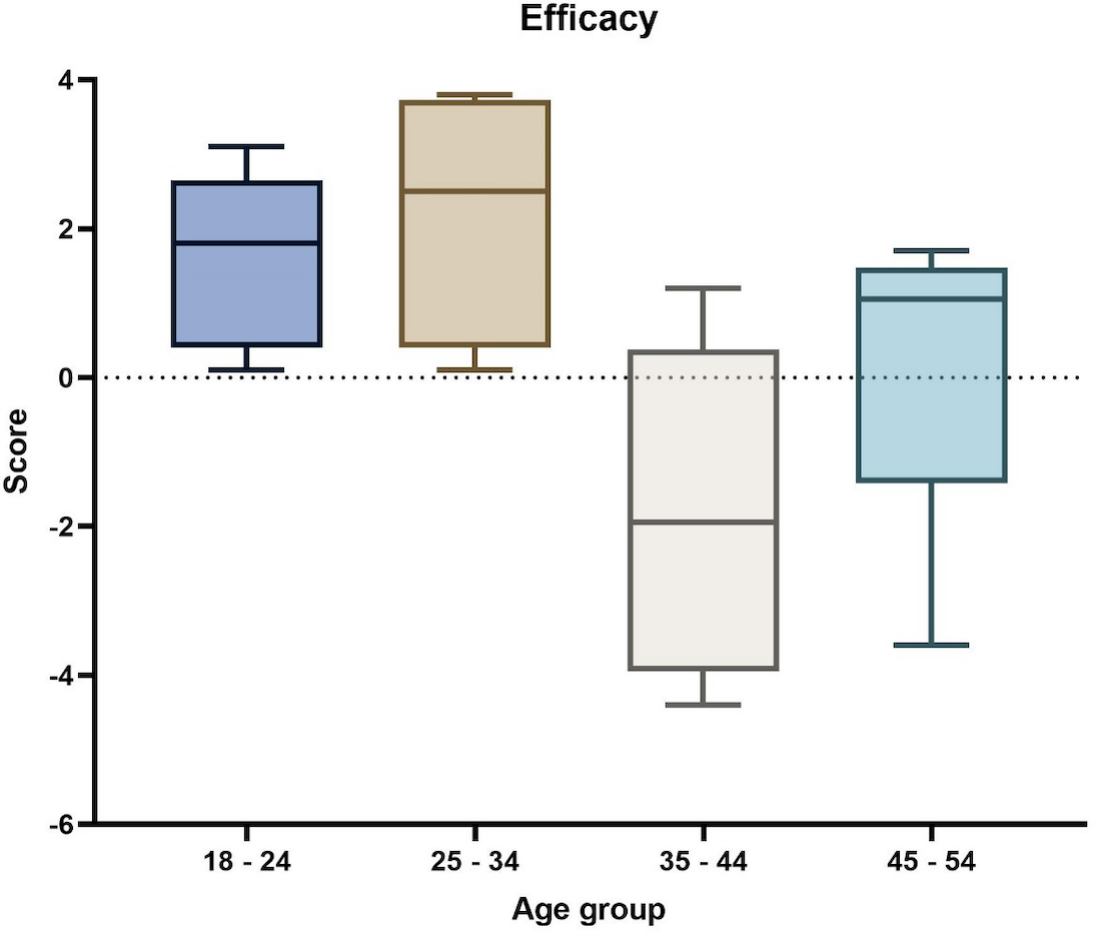
Incremental lift in perceived efficacy of COVID-19 vaccines by age group across 5 Brand Lift Studies in Mexico, 2021‐2022.

The comparison by age group was conducted, and the mean and SD are presented in Table 5. A statistically significant difference was found in the mean efficacy score (*P*=.005) between the age groups, as shown in Figure 3.

The comparison by sex was conducted for each campaign, and the mean and SD are presented in Table 6. No statistically significant differences were found in the scores of any of the items between the sex groups (all *P*≥.06).

An evaluation was conducted to assess the association between the probability of behavior change by the campaign. A statistically significant association was found between the probability of standard ad recall of 90% or more (*P*=.006) and 80% or more (*P*=.03) with the campaign; all the evaluations are presented in Table 7.

An evaluation was conducted to assess the association between the probability and age group. No statistically significant association was found between the probabilities of the items and the age groups (*P*=.10 or more); all the evaluations are presented in Table 8.

**Table 8.** Association between probability of behavior change (≥80% or ≥90% chance of Brand Lift) and age group in 5 Brand Lift Studies of COVID-19 vaccination campaigns in Mexico, 2021‐2022.

Probability of Brand Lift	Age group (years), n (%)						*P* value^a^
	18‐24	25‐34	35‐44	45‐54	55‐64	65+	
Standard ad recall of 90% or more							.25
Yes	4 (44.4)	2 (22.2)	1 (11.1)	0 (0)	0 (0)	0 (0)	
No	5 (55.6)	7 (77.8)	8 (88.9)	8 (100)	2 (100)	2 (100)	
Standard ad recall of 80% or more							.30
Yes	1 (11.1)	1 (11.1)	3 (33.3)	0 (0)	0 (0)	1 (50)	
No	8 (88.9)	8 (88.9)	6 (66.7)	8 (100)	2 (100)	1 (50)	
Importance of 90% or more							.10
Yes	3 (37.5)	5 (62.5)	2 (25)	0 (0)	0 (0)	0 (0)	
No	5 (62.5)	3 (37.5)	6 (75)	8 (100)	2 (100)	2 (100)	
Importance of 80% or more							.43
Yes	2 (25)	0 (0)	2 (25)	0 (0)	0 (0)	0 (0)	
No	6 (75)	8 (100)	6 (75)	8 (100)	2 (100)	2 (100)	
Safety of 90% or more							.51
Yes	2 (22.2)	2 (22.2)	0 (0)	0 (0)	0 (0)	0 (0)	
No	7 (77.8)	7 (77.8)	9 (100)	8 (100)	2 (100)	2 (100)	
Safety of 80% or more							.18
Yes	0 (0)	3 (33.3)	0 (0)	0 (0)	0 (0)	0 (0)	
No	9 (100)	6 (66.7)	9 (100)	8 (100)	2 (100)	2 (100)	
Efficacy of 90% or more							.28
Yes	0 (0)	2 (33.3)	0 (0)	0 (0)	0 (0)	0 (0)	
No	6 (100)	4 (66.7)	6 (100)	6 (100)	0 (0)	0 (0)	
Efficacy of 80% or more							.57
Yes	2 (33.3)	1 (16.7)	0 (0)	0 (0)	0 (0)	0 (0)	
No	4 (66.7)	5 (83.3)	6 (100)	6 (100)	0 (0)	0 (0)	
Side effects of 90% or more							
Yes	0 (0)	0 (0)	0 (0)	0 (0)	0 (0)	0 (0)	
No	1 (100)	1 (100)	1 (100)	0 (0)	0 (0)	0 (0)	
Side effects of 80% or more							
Yes	0 (0)	0 (0)	0 (0)	0 (0)	0 (0)	0 (0)	
No	1 (100)	1 (100)	1 (100)	0 (0)	0 (0)	0 (0)	

a Fisher exact test.

An evaluation was conducted to assess the association between the probability and sex. No statistically significant association was found between the probabilities of the items and sex (*P*=.11 or more); all the evaluations are presented in Table 9.

**Table 9.** Association between probability of behavior change (≥80% or ≥90% chance of Brand Lift) and sex in 5 Brand Lift Studies of COVID-19 vaccination campaigns in Mexico, 2021‐2022.

Probability of Brand Lift	Sex		*P* value^a^
	Male, n (%)	Female, n (%)	
Standard ad recall of 90% or more			.42
Yes	2 (11.1)	5 (23.8)	
No	16 (88.9)	16 (76.2)	
Standard ad recall of 80% or more			>.99
Yes	3 (16.7)	3 (14.3)	
No	15 (83.3)	18 (85.7)	
Importance of 90% or more			.26
Yes	3 (16.7)	7 (38.9)	
No	15 (83.3)	11 (61.1)	
Importance of 80% or more			>.99
Yes	2 (11.1)	2 (11.1)	
No	16 (88.9)	16 (88.9)	
Safety of 90% or more			.11
Yes	0 (0)	4 (19)	
No	18 (100)	17 (81)	
Safety of 80% or more			.59
Yes	2 (11.1)	1 (4.8)	
No	16 (88.9)	20 (95.2)	
Efficacy of 90% or more			>.99
Yes	1 (8.3)	1 (8.3)	
No	11 (91.7)	11 (91.7)	
Efficacy of 80% or more			>.99
Yes	2 (11.1)	1 (8.3)	
No	10 (83.3)	11 (91.7)	
Side effects of 90% or more			
Yes	0	0 (0)	
No	0	3 (100)	
Side effects of 80% or more			
Yes	0	0 (0)	
No	0	3 (100)	

a Fisher exact test.

## Discussion

### Overview

To our knowledge, this is the first study to use experimental BLS data to evaluate vaccine communication in Mexico, providing a scalable model for assessing digital health interventions in other LMICs. This study evaluated the effectiveness of 5 large-scale social media advertising campaigns on COVID-19 vaccination in Mexico, using Meta’s BLS to assess standard ad recall and perceptions of vaccine importance, safety, efficacy, and side effects. The findings demonstrate that digital campaigns can generate measurable shifts in awareness and attitudes, although the magnitude and consistency of these effects varied across campaigns and subgroups. These results partially confirm our hypothesis that targeted campaigns, particularly those directed at younger populations and women, would generate stronger positive perceptual shifts.

### Principal Findings

Standard ad recall increased significantly in 4 of the 5 campaigns, with the women-focused campaign (aged 18‐44 years in major cities) achieving the largest effect (+11.6 percentage points compared to control). Perceived importance of vaccination improved in 3 of the 4 campaigns where it was measured, with incremental lifts ranging from +1.4 to +3.2 points. Perceptions of safety showed modest but positive gains in 2 campaigns (up to +2.5 points), while efficacy perceptions improved only marginally (≤1.1 points). Concerns about side effects did not change significantly. These results indicate that recall and importance were the most responsive outcomes, whereas safety and efficacy perceptions were more resistant to change.

### Differences Between Campaigns

Targeted campaigns produced stronger effects than general population campaigns. In particular, the women-focused campaign not only achieved the highest recall but also showed significant improvements in safety perceptions. In contrast, Campaign 5, which addressed doubts about booster effectiveness, did not produce significant changes in recall or perceptions. This suggests that audience segmentation and message framing are critical determinants of campaign success [3,4].

### Subgroup Analysis

Younger audiences (18‐34 years) consistently exhibited stronger positive shifts in recall and importance compared to older groups. For example, in Campaign 3, recall increased by +5.0 points among men aged 18‐24 years, while no significant change was observed in older age groups. No consistent differences were observed by sex, except in the women-targeted campaign, where effects were concentrated among the intended audience. These findings align with prior evidence that younger, digitally native populations are more responsive to online health interventions [2,3].

### Interpretation in Context

Our results are consistent with international evidence showing that digital advertising can shift health-related attitudes [3,4]. The stronger effects observed among younger audiences and in targeted campaigns suggest that segmentation strategies enhance the effectiveness of digital health communication [2]. However, the limited changes in perceptions of efficacy and side effects highlight the challenges of addressing entrenched concerns through short-format digital ads alone. These dimensions may require complementary strategies, such as longer-form educational content or integration with offline communication [9].

### Limitations

This study has several limitations. First, outcomes were limited to recall and perceptions; no direct behavioral measures (eg, vaccination uptake) were available. Thus, while perceptual shifts are important precursors to behavior, they cannot be equated with actual vaccination. Although the BLS do not capture behavioral end points such as vaccination uptake, these attitudinal outcomes are therefore considered health-related because they influence individuals’ likelihood of seeking vaccination and engaging with preventive health services.

Second, although randomization at the user ID level ensured balance between test and control groups, the data available to researchers were anonymized and aggregated, with only age group and sex provided in disaggregated form. As a result, we could not control for additional individual-level covariates (eg, prior exposure to vaccine information and baseline attitudes), which may have introduced residual confounding in subgroup analyses. Third, small sample sizes in some subgroups reflect the BLS survey design, where each respondent answers only 1 question, and should be interpreted with caution. Finally, the findings represent the digital population and may not generalize to individuals without internet or social media access. Our study includes a lack of individual participant data for conducting a cost-benefit evaluation and insufficient information on the number of vaccines administered in Mexico during the campaigns to assess their impact on the vaccination program. It is important to note that not all segments of the Mexican population use digital platforms. Therefore, the findings of this study primarily represent the digital population and may not be generalizable to those who are not connected to or active on social media platforms.

### Implications

Despite these limitations, this study provides novel evidence on the role of social media in vaccine communication in a middle-income country. The results indicate that digital campaigns can effectively increase standard ad recall and improve perceptions of vaccine importance and safety, particularly when targeted to specific audiences. These findings reinforce that audience segmentation—especially toward younger adults and urban women—enhances the effectiveness of digital health messaging compared with broad, non-targeted approaches.

This study offers an innovative application of Meta’s experimental BLS data to evaluate large-scale, real-world vaccination campaigns in Latin America—a setting where such experimental evidence remains scarce. By demonstrating that different demographic groups respond differently to campaign messages, the study provides practical insights for designing more efficient communication strategies.

These findings have several implications for public health practice. First, digital vaccination campaigns should prioritize precise audience segmentation to maximize impact while optimizing resources. Second, because BLS provide rapid, experiment-based feedback, they can be incorporated into real-time monitoring systems that allow authorities to adjust message framing, creative content, or targeting while campaigns are still active. Third, combining digital strategies with community-based interventions may help reinforce attitudinal changes and extend impact beyond online spaces.

For policymakers, this study illustrates how experimental digital metrics can complement traditional epidemiological surveillance. Future digital vaccination efforts in Mexico and other LMICs should integrate attitudinal indicators (eg, confidence and perceived safety) with real-world vaccination uptake from national immunization systems. Linking shifts in vaccine acceptance with the number of doses administered during campaign periods would provide a more robust measure of true impact and generate evidence to guide investment in digital public health interventions.

Overall, this study contributes to the field by offering a replicable framework for measuring attitudinal effects of social media advertising using experimental causal methods seldom applied in LMIC settings. In practical terms, the findings demonstrate how digital platforms can complement traditional communication strategies by improving awareness and perceptions in key demographic groups, guiding authorities in resource allocation, message tailoring, and integration of digital analytics into broader vaccination efforts.

### Conclusions

This study provides new evidence on the effectiveness of large-scale social media campaigns to influence vaccine-related perceptions in a Latin American middle-income country. Across 5 Meta BLS, digital advertising significantly increased standard ad recall and improved perceptions of vaccine importance and safety, particularly among younger adults and women in urban areas.

Our findings demonstrate that audience segmentation and message targeting enhance the impact of digital communication, suggesting that health authorities and organizations can use social media strategically to strengthen vaccine confidence. Although the study did not measure vaccination behavior directly, perceptual shifts represent important precursors to uptake and highlight the potential of digital platforms to complement traditional public health strategies.

This work contributes to the field by applying experimental BLS for LMICs data to evaluate real-world public health communication, where evidence on digital health interventions remains limited. These results can inform the design of future digital campaigns, guide resource allocation, and support the integration of digital analytics into broader immunization efforts. Future research should link digital engagement metrics with epidemiological data to assess downstream behavioral outcomes.

There is no doubt that there is still much work to be done, but it is of great importance for health specialists to continue collaborating and engaging audiences in digital media. By creating experts in the dissemination of medical information and generating behavioral changes, we can improve the health of the population. However, to identify the best strategies, it is essential to generate evidence to use as a basis for creating, designing, and evaluating successful campaigns that lead to real behavioral changes in health worldwide.

## Abbreviations

BLS: Brand Lift Studies
CIOMS: Council for International Organizations of Medical Sciences
LMIC: low- and middle-income country
SBCC: Social and Behavior Change Communication
WHO: World Health Organization

## References

[1] Brewer NT, Chapman GB, Rothman AJ, Leask J, Kempe A (2017). Increasing vaccination: putting psychological science into action. Psychol Sci Public Interest.

[2] Andreas M, Iannizzi C, Bohndorf E (2022). Interventions to increase COVID-19 vaccine uptake: a scoping review. Cochrane Database Syst Rev.

[3] Athey S, Grabarz K, Luca M, Wernerfelt N (2023). Digital public health interventions at scale: the impact of social media advertising on beliefs and outcomes related to COVID vaccines. Proc Natl Acad Sci U S A.

[4] Evans WD, Abroms LC, Broniatowski D (2022). Digital media for behavior change: review of an emerging field of study. Int J Environ Res Public Health.

[5] (2024). Encuesta nacional sobre disponibilidad y uso de tecnologías de la información en los hogares (ENDUTIH) [Website in Spanish]. Instituto Federal de Telecomunicaciones.

[6] Hansen RK, Baiju N, Gabarron E (2023). Social media as an effective provider of quality-assured and accurate information to increase vaccine rates: systematic review. J Med Internet Res.

[7] Ferreira-Silva SN, Soares MEM, Vasconcelos R (2024). COVID-19 vaccine hesitancy: meaning relations between responses in an epidemiological study and twitter messages. Vaccine (Auckl).

[8] Mascherini M, Nivakoski S (2022). Social media use and vaccine hesitancy in the European Union. Vaccine (Auckl).

[9] Ahmed S, Rasul ME, Cho J (2022). Social media news use induces COVID-19 vaccine hesitancy through skepticism regarding its efficacy: a longitudinal study from the United States. Front Psychol.

[10] Liebermann E, Kornides M, Matsunaga M (2025). Use of social media and its influence on HPV vaccine hesitancy: US National Online Survey of mothers of adolescents, 2023. Vaccine (Auckl).

[11] Jordan S, Böttger SJ, Zinn S (2025). The persuasiveness of different sources of information on the decision to vaccinate. A cross-sectional study in Germany during the pandemic at the turn of the year 2021/2022. PLoS One.

[12] Nelson V, Bashyal B, Tan PN, Argyris YA (2024). Vaccine rhetoric on social media and COVID-19 vaccine uptake rates: a triangulation using self-reported vaccine acceptance. Soc Sci Med.

[13] (2016). Data policy [Website in Spanish]. Facebook.

[14] About brand lift tests. Meta Business Help Centre.

[15] About A/B testing. Meta.

[16] About lift and holdouts in facebook advertising tests. Meta.

[17] Gordon BR, Zettelmeyer F, Bhargava N, Chapsky D (2019). A comparison of approaches to advertising measurement: evidence from Big Field Experiments at Facebook. Mark Sci.

[18] Journal Article Reporting Standards: Quantitative Research (JARS–Quant) APA Style. American Psychological Association.

